# Object Detection and Classification Framework for Analysis of Video Data Acquired from Indian Roads

**DOI:** 10.3390/s24196319

**Published:** 2024-09-29

**Authors:** Aayushi Padia, Aryan T. N., Sharan Thummagunti, Vivaan Sharma, Manjunath K. Vanahalli, Prabhu Prasad B. M., Girish G. N., Yong-Guk Kim, Pavan Kumar B. N.

**Affiliations:** 1 Department of DSAI, Indian Institute of Information Technology, Dharwad 580009, India; 21bds002@iiitdwd.ac.in (A.P.); 21bds006@iiitdwd.ac.in (A.T.N.); 21bds061@iiitdwd.ac.in (S.T.); 21bds070@iiitdwd.ac.in (V.S.); manjunathkv@iiitdwd.ac.in (M.K.V.); 2 Department of CSE, Indian Institute of Information Technology, Dharwad 580009, India; prabhuprasad1990@gmail.com (P.P.B.M.); girish.anit@gmail.com (G.G.N.); 3 Department of Computer Engineering, Sejong University, Seoul 05006, Republic of Korea; ykim@sejong.ac.kr; 4 Computer Science and Engineering Group, Indian Institute of Information Technology, Sri City 517646, India

**Keywords:** YOLOv8, scalable, memory efficient, weather conditions, dense traffic, sensors, intelligent transportation systems (ITSs), intelligent vehicles (IVs), artificial intelligence (AI)

## Abstract

Object detection and classification in autonomous vehicles are crucial for ensuring safe and efficient navigation through complex environments. This paper addresses the need for robust detection and classification algorithms tailored specifically for Indian roads, which present unique challenges such as diverse traffic patterns, erratic driving behaviors, and varied weather conditions. Despite significant progress in object detection and classification for autonomous vehicles, existing methods often struggle to generalize effectively to the conditions encountered on Indian roads. This paper proposes a novel approach utilizing the YOLOv8 deep learning model, designed to be lightweight, scalable, and efficient for real-time implementation using onboard cameras. Experimental evaluations were conducted using real-life scenarios encompassing diverse weather and traffic conditions. Videos captured in various environments were utilized to assess the model’s performance, with particular emphasis on its accuracy and precision across 35 distinct object classes. The experiments demonstrate a precision of 0.65 for the detection of multiple classes, indicating the model’s efficacy in handling a wide range of objects. Moreover, real-time testing revealed an average accuracy exceeding 70% across all scenarios, with a peak accuracy of 95% achieved in optimal conditions. The parameters considered in the evaluation process encompassed not only traditional metrics but also factors pertinent to Indian road conditions, such as low lighting, occlusions, and unpredictable traffic patterns. The proposed method exhibits superiority over existing approaches by offering a balanced trade-off between model complexity and performance. By leveraging the YOLOv8 architecture, this solution achieved high accuracy while minimizing computational resources, making it well suited for deployment in autonomous vehicles operating on Indian roads.

## 1. Introduction

Autonomous vehicles have the potential to change transportation networks globally, providing advantages like heightened security, diminished gridlock, and enhanced mobility. However, achieving this goal requires overcoming formidable technical obstacles mainly related to guaranteeing the dependability and safety of autonomous navigation systems. This challenge revolves around real-time obstacle detection and classification, which is essential to allowing autonomous vehicles to perceive and navigate through dynamic and complex environments safely. With a focus on addressing the particular challenges posed by Indian roads, this research presents a novel machine learning-based system designed specifically for real-time obstacle detection and classification in autonomous vehicles.

While various object detection models, such as YOLOv7, YOLOv5, and Fast R-CNN, have demonstrated success in obstacle detection tasks, they are primarily designed and trained on datasets that do not fully capture the unique and unpredictable conditions prevalent on Indian roads. Indian roads are characterized by a wide range of obstacles, including fluctuating traffic patterns, erratic driver behavior, varying environmental conditions, and infrastructural inconsistencies. Existing models often struggle to maintain accuracy and reliability in such conditions, as they are not specifically tailored to handle the diverse and chaotic nature of this environment.

Moreover, research on autonomous driving systems has largely focused on well-structured roads found in Western and developed nations, leaving a significant gap in the understanding and optimization of such systems for developing countries like India. The lack of dedicated research and datasets designed for Indian roads further compounds the challenge, as current models may not generalize well to the intricate road scenarios encountered here.

Using the YOLOv8 object detection model, which is well-known for its effectiveness and precision in recognizing objects in pictures and video streams, this system seeks to offer strong and dependable obstacle detection capabilities that can function in real time. By precisely identifying and categorizing obstacles found on Indian roads, the system aims to improve safety and situational awareness in autonomous vehicle navigation. The Indian roads dataset, which captures the varied and difficult road conditions common in India, such as fluctuating traffic patterns, environmental factors, driver uncertainty, and infrastructure limitations, is used to analyze real-world scenarios in order to achieve this.

This work’s principal contributions are as follows:

- Tailored solution for Indian road conditions: Unlike existing object detection models, which are typically optimized for structured road environments found in developed regions, this study presents a novel approach specifically designed to address the unique challenges of Indian roads. These include irregular driving patterns, diverse weather conditions, and highly dynamic traffic situations. By using a dataset that captures these specific nuances, the model is able to outperform existing methods in scenarios that are underexplored by conventional object detection systems.
- Effective YOLOv8 real-time object detection: While prior models like YOLOv7 and YOLOv5 have demonstrated strong object detection performance, this paper leverages the YOLOv8 deep learning architecture for its superior balance between accuracy and computational efficiency. This contribution distinguishes itself by minimizing computational overhead while maintaining real-time detection capabilities, making it feasible for deployment in resource-constrained environments like autonomous vehicles in India, where low-latency processing is critical.
- Comprehensive evaluation and high precision: Unlike most existing research, which is often tested under limited or controlled conditions, this study rigorously evaluates the proposed model in diverse real-world scenarios, including adverse weather and complex traffic patterns. With a very good accuracy across all tested scenarios and a precision of 0.65 for 35 different object classes, the model demonstrates robustness and reliability that surpasses many traditional approaches, which tend to specialize in fewer object classes or fail in challenging environments.

## 2. Related Study

### 2.1. Object Detection in Autonomous Vehicles: Status and Open Challenges

Deep learning-based two-stage and single-stage detectors are the two primary methods used in object detection. Strong but computationally demanding models are produced by two-stage detectors, such as R-CNN, Fast R-CNN, and Faster R-CNN, which employ a region proposal stage followed by object classification. Single-stage detectors prioritize speed over complexity, performing localization and classification in a single pass. Examples of such detectors are YOLO, SSD, EfficientNet, and RetinaNet [1].

R-CNN, the precursor to modern object detection, had problems with accuracy and efficiency. By incorporating feature extraction for the full image, Fast R-CNN enhanced and Faster R-CNN further expedited detection with a specialized region proposal network. In contrast, YOLO, especially YOLOv2 and YOLOv3, revolutionized object detection with single-pass detection, greatly improving inference speed along with enhancement of accuracy by normalization and anchor boxes.

### 2.2. YOLO Versions Architecture: Review

The evolution of the YOLO (You Only Look Once) series, spanning YOLOv1 to YOLOv8, has significantly influenced the field of object detection in computer vision. Each iteration has introduced innovative architectural designs and features, reshaping the landscape of real-time object detection. This review provides a detailed examination of the architectural evolution, key features, and performance metrics of each YOLO version [2].

Single-stage object detection was introduced in YOLOv1, the predecessor to YOLO. Using anchor boxes of various sizes and Darknet backbones, YOLOv2 and YOLOv3 improved the architecture. YOLOv5 employed the EfficientDet design, while YOLOv4 embraced CSPNet. YOLOv6 used EfficientNet-L2 to maximize efficiency, and YOLOv7 used spatial pyramid pooling (SPP) to increase speed, with improved Bottleneck structures and anchor-free detection. YOLOv8 made even more progress.

Each YOLO version showcases unique architectural elements. Darknet backbones, characterized by convolutional layers and 1 × 1 convolutions for parameter reduction, formed the foundation of YOLO architectures. Batch normalization and focal loss functions were integrated to enhance model regularization and address challenges in detecting small objects. YOLOv4 introduced CSPNet, a modified ResNet architecture optimized for object detection tasks, while YOLOv5 adopted EfficientDet architecture, leveraging EfficientNet-L2 for improved computational efficiency. YOLOv7 introduced the focused loss function to prioritize challenging examples, enhancing model robustness. YOLOv8 [3] made significant modifications to Bottleneck structures and embraced anchor-free detection. Further developments in YOLOv8 [4,5,6] promise to unlock new possibilities in object detection.

### 2.3. YOLOv8-Based Visual Detection of Road Hazards: Potholes, Sewer Covers, and Manholes

In order to tackle the crucial issue of identifying road hazards including potholes, sewer covers, and manholes [7], this research explores the use of YOLOv8, a sophisticated object detection model [8]. It thoroughly analyzes the architecture of YOLOv8 [9] and compares it to previous versions such as YOLOv7 and YOLOv5.

From a methodological perspective, this study carefully describes the architectural details of YOLOv8, clarifying its fundamental elements, including the detecting head, backbone characteristics, and the addition of YOLOv8-Seg for semantic segmentation. It carefully outlines the several facets of training a model, including selecting the right dataset, applying preprocessing steps [10], and using training approaches that maximize model performance [11].

### 2.4. DC-YOLOv8: Small Size Object Detection Algorithm Based on Camera Sensor

The DC-YOLOv8 algorithm [12] is developed to address challenges in accurately detecting small objects in complex scenes using camera sensors. It incorporates several enhancements:

- Small target identification: DC-YOLOv8 addresses challenges in detecting small targets often overshadowed by larger objects or complex scenes [13].
- Algorithmic improvements: It introduces the MDC Module, leveraging depth separable convolution, max-pooling, and refined feature fusion to preserve context information effectively. The DC Module integrates DenseNet and VOVNet concepts for deeper network architecture, minimizing information loss from large objects.
- Experimental validation: DC-YOLOv8 undergoes rigorous evaluation on datasets like Visdrone [14], Pascal VOC2007 [15], and Tinyperson [16], demonstrating superior performance over classical algorithms such as YOLOv3, YOLOv5, and YOLOv7, especially in detecting small targets.

This study concludes that DC-YOLOv8 offers significant advancements in both accuracy and speed for small object detection in complex scenes.

### 2.5. StrongSORT: Make DeepSORT Great Again

“StrongSORT” is an upgraded version of the traditional DeepSORT [17] tracker, designed to enhance its performance in multiple object tracking (MOT) tasks. The enhancements include the integration of new modules and inference techniques, which collectively improve the tracking accuracy and robustness of the system. Additionally, two lightweight algorithms named AFLink and GSI are introduced to address specific challenges related to missing association and missing detection [18].

### 2.6. Multi-Modal 3D Object Detection in Autonomous Driving: A Survey

Multi-modal 3D object detection [19] is a crucial technology for autonomous vehicles to perceive their surroundings. It combines data from radar, LiDAR, and cameras, which provides a comprehensive view. Radar performs better in poor weather but has lower resolution, while LiDAR is better at measuring distance but struggles with sparse data.

Researchers use datasets like KITTI [20] and Waymo [21] to benchmark and improve detection algorithms under various conditions. A key challenge is how to best fuse data from these different sensors. Common fusion techniques include region-of-interest (RoI), voxel, and point-level fusion. Additionally, cost-effective detection methods are necessary. Techniques like knowledge distillation from LiDAR to camera data are being explored to achieve good detection accuracy at a lower cost.

Finally, the scarcity of real-world data is a hurdle. Researchers are looking into synthetic datasets and techniques like photorealistic rendering to bridge the gap and create more training data. Overall, multi-modal 3D object detection [22] is a rapidly developing field with the potential to revolutionize autonomous driving.

### 2.7. Monocular Camera-Based Complex Obstacle Avoidance via Efficient Deep Reinforcement Learning

The literature introduces a framework for mobile robot navigation using only a monocular RGB camera [23]. It utilizes a pseudo-laser, combining depth and semantic information, alongside a Feature Extraction Guidance module and deep reinforcement learning. The framework demonstrates a solution for monocular camera-based obstacle avoidance in mobile robotics [24].

### 2.8. IDD: A Dataset for Exploring Problems of Autonomous Navigation in Unconstrained Environments

This paper introduces the Indian Driving Dataset (IDD) [25], aiming to address the limitations of existing datasets for autonomous navigation by focusing on unstructured driving environments. Unlike datasets that primarily cover structured environments with well-defined infrastructure and traffic rules [26], IDD captures the complexity of Indian roads, including diverse traffic participants, ambiguous road boundaries, and varying environmental conditions.

The dataset comprises 10,004 images annotated with 34 classes, collected from 182 drive sequences in Indian cities. IDD expands the label set compared to benchmarks like Cityscapes, reflecting the unique characteristics of Indian road scenes. This paper suggests IDD is an ideal resource for addressing new research problems such as domain adaptation, few-shot learning, and behavior prediction in unstructured road scenes, emphasizing the need for larger and more diverse datasets for autonomous systems.

### 2.9. NITCAD—Developing an Object Detection, Classification, and Stereo Vision Dataset for Autonomous Navigation in Indian Roads

The literature introduces the NITCAD dataset, designed specifically for developing autonomous navigation systems suited to Indian road conditions [27]. The dataset comprises both RGB and stereo images collected using cameras mounted on a vehicle traversing rural and urban roads in Kerala, India. This comprehensive dataset includes two main components: the NITCAD object dataset and the NITCAD stereo vision dataset. The former focuses on object classification and detection, while the latter aims to estimate depth for navigation purposes.

For evaluating the performance of autonomous navigation systems, the authors employed various deep learning architectures on the NITCAD object dataset. These architectures were tested for classification accuracy, precision, and recall across six different classes, which are auto-rickshaw, bus, car, pedestrian, truck, two-wheeler, and van [28]. Results showed variations in performance among architectures, with Xception demonstrating the highest precision and recall for most classes.

Object detection was evaluated using Faster R-CNN, achieving an accuracy of 89.4%. Precision and recall values varied across classes, with each architecture demonstrating strengths and weaknesses in detecting specific objects.

### 2.10. Faster RCNN-Based Robust Vehicle Detection Algorithm for Identifying and then Classifying Vehicles

This research proposes a new method for real-time vehicle detection using deep learning. It improves upon Faster R-CNN [29] by incorporating techniques like Soft NMS and using modified base network architectures like MobileNetV3 and a tweaked VGG16. The model is trained on a custom dataset with heavily occluded vehicles, similar to real-world situations. It outperforms older Faster R-CNN versions and competes well with state-of-the-art methods.

### 2.11. Single Shot Multi-Box Detector Algorithm over Fast R-CNN: An Ingenious Technique for Increasing Object Detection Classification Accuracy

This review examines advancements in object detection algorithms, with a focus on achieving higher classification accuracy. Researchers have proposed various methods to address precision challenges. Some approaches include Kumar et al.’s (2017) [30] pivot-aware target location for Faster R-CNN, which boosted precision by 2.5%, and the Single Shot Multi-box Detector (SSD) [31] introduced by Xiang et al. (2018) [32] as a fast and effective object recognition method.

The latter half of the review emphasizes the superiority of the recent SSD algorithm compared to traditional methods like Fast R-CNN. SSD achieves better accuracy through its use of skip pooling to combine contextual data.

## 3. Proposed Work

### 3.1. Data Acquisition

For this research, the Dats_2022 [33] dataset was carefully curated to specifically address the unique challenges of the Indian road environment. Pedestrian-related classes are particularly critical in Indian road settings, and the dataset emphasizes these classes to enhance accuracy in object detection and classification. With a size of 10 GB, the dataset contains 2048 training images and 802 test images, offering a diverse representation of urban landscapes, vehicular traffic, and pedestrian activity characteristic of Indian streets.

**Figure 1:**
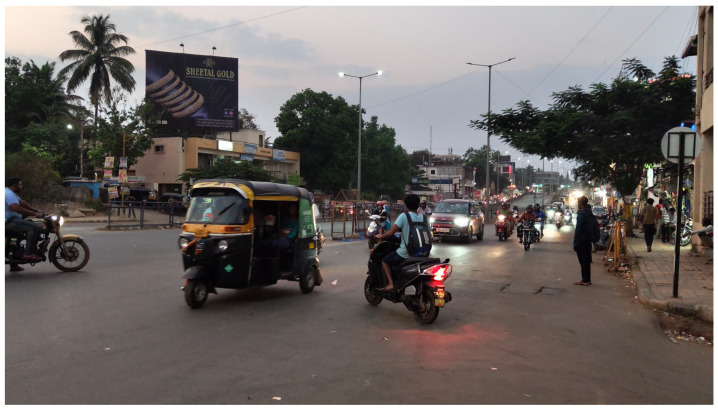
Data sample.

The dataset’s footage was collected from Indian roads, and its class labels reflect objects that are more commonly encountered in this setting than in others, making it an ideal fit for the research. Some of the key classes include rickshaws, tractors, cattle, and carts, which are prevalent on Indian roads in many regions. These factors contribute to improved performance when addressing the complexities of Indian traffic. Figure 1 provides an example image that captures the essence of the dataset.

**Figure 2:**
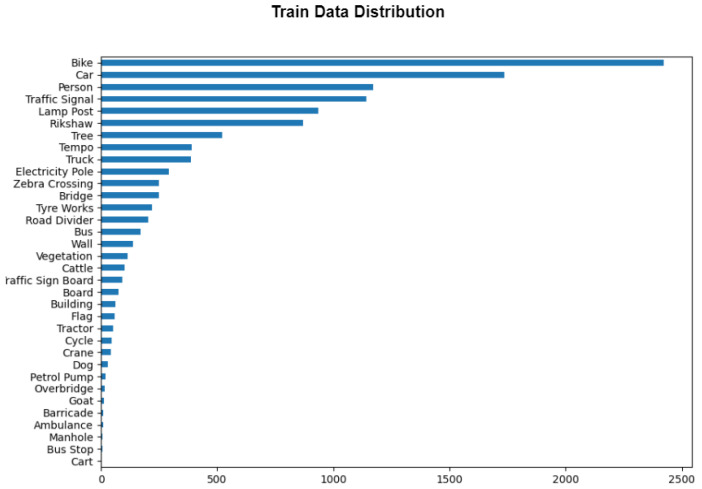
Training data distribution.

**Figure 3:**
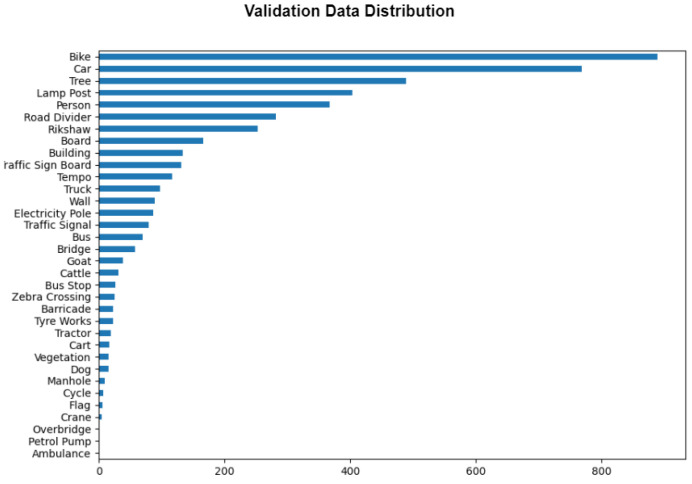
Validation data distribution.

The data collection process was comprehensive, drawing from various sources to accurately reflect the diversity of Indian road conditions. Initially, the distribution of the Dats_2022 dataset was found to be skewed, as shown in Figure 2 and Figure 3, which display the training and validation data distributions. To address this imbalance, additional images were sourced from Roboflow [34] for classes that were underrepresented or nearly absent in the original dataset. These underrepresented classes include objects such as manholes, ambulances, petrol pumps, and overbridges. The new images were provided in a YOLOv8-trainable format, which allowed them to be seamlessly integrated into the dataset.

This augmentation resulted in a total of 4296 training images [35], comprising 3451 images with objects and 845 background images. The dataset spans various road types, including urban streets, highways, and rural roads, as well as different environmental conditions like variable lighting, weather, and traffic densities.

The dataset features 35 distinct labels, covering a wide range of objects, from vehicles such as cars and bikes to pedestrians, and environmental elements like traffic signs. The full list of labels includes the following: Traffic signal, Lamp post, Zebra crossing, Bike, Car, Rickshaw, Tyre works, Tree, Tractor, Cattle, Vegetation, Electric pole, Building, Wall, Person, Bus, Bridge, Road Divider, Traffic signboard, Flag, Crane, Cycle, Dog, Truck, Overbridge, Manhole, Bus stop, Barricade, Petrol pump, Ambulance, Goat, Cart, and Background. Notably, the most common labels in the images are Car, Bike, and Rickshaw.

### 3.2. Preprocessing

Recognizing the pivotal role of data preprocessing in fortifying model robustness and enhancing generalization capabilities, this methodology prioritizes the application of a suite of sophisticated data augmentation techniques:

- Annotation: Annotators were trained to accurately label objects in the images using bounding boxes, ensuring consistency and accuracy across the dataset.
- Augmentation: To increase dataset diversity and improve model generalization, augmentation techniques were applied. This includes geometric transformations such as rotation, scaling, and flipping. They ensure that the model learns to recognize objects from different angles, distances, and orientations, which is especially important in the context of autonomous vehicles navigating dynamic environments where obstacles may appear in various forms.
  Photometric transformations, on the other hand, like brightness adjustment and color augmentation, help the model become robust to changes in lighting conditions and visual appearance, such as those caused by weather or time of day. These augmentations introduce variability into the training data, simulating the diverse conditions the model will encounter in deployment. By training the model on these augmented data, we reduce the risk of overfitting and improve its performance on unseen data, ensuring better detection and classification accuracy in unpredictable environments like Indian roads.
- Data splitting: The dataset was divided into training, validation, and testing sets to facilitate model training and evaluation. Care was taken to ensure that each set represented a balanced distribution of classes to prevent bias during training.

**Figure 4:**
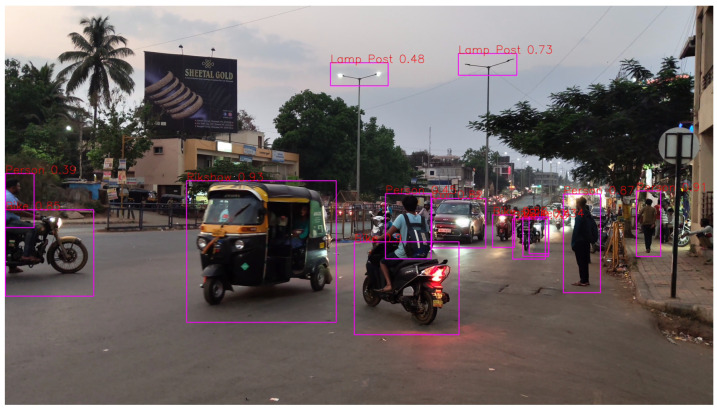
Preprocessed data.

By subjecting the dataset to these transformative operations, the aim is to imbue this model with a heightened adaptability to the myriad of real-world scenarios encountered on Indian roads. An example of the preprocessed data is shown in Figure 4 to better understand the process.

### 3.3. Feature Engineering and Model Architecture

**Figure 5:**
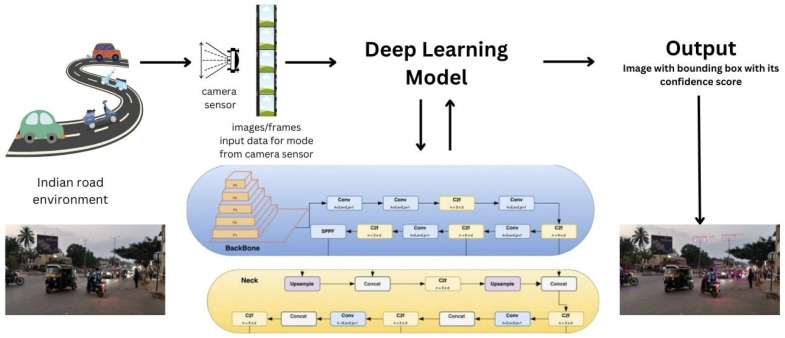
Dataflow pipeline.

The feature engineering and model architecture involve a streamlined dataflow pipeline designed for real-time obstacle detection in autonomous vehicles, as shown in Figure 5. The process begins with a camera capturing live images of the surroundings. These images are then fed into the deep learning model, specifically YOLOv8, which processes the input to detect and classify objects present in the scene. The model extracts relevant features from the images, such as shapes, colors, and textures, allowing it to identify different types of obstacles with high accuracy. Once detected and classified, the model outputs the results.

1. Input data acquisition: The input data are acquired from a camera sensor, which could be mounted on a device or integrated into a surveillance system. The camera captures images or video streams of the scene, providing the raw data for object detection.
2. Frame extraction (video to frames): For video inputs, the video stream is processed to extract individual frames using video processing techniques. Libraries such as OpenCV are commonly used for this task, allowing seamless conversion of video streams into a series of frames.
3. Object detection and classification: The core of the dataflow pipeline is the YOLOv8 Nano model, which is utilized for real-time object detection and classification. YOLOv8 Nano is a lightweight variant of the YOLOv8 architecture, optimized for resource-constrained environments while maintaining high accuracy. The YOLOv8 architecture employs a single neural network to predict bounding boxes and class probabilities directly from full images in one evaluation. This approach enables efficient real-time inference by eliminating the need for region proposal networks or multiple stages of processing.
4. YOLOv8 architecture: The YOLOv8 [36] architecture builds upon the previous versions of YOLO (You Only Look Once) models, introducing improvements in accuracy and efficiency. The backbone network of YOLOv8, typically based on Darknet, extracts features from the input images. This is crucial for detecting objects in diverse and cluttered environments typical of Indian roads, where road scenes can vary widely in terms of vehicle types, pedestrian density, and environmental conditions. Detection layers are responsible for predicting bounding boxes and class probabilities at multiple scales.
  YOLOv8 uses PANet for better multi-scale feature fusion. PANet enhances the model’s ability to detect objects at various scales and improves its robustness to variations in object size and appearance due to different lighting and environmental conditions. This is achieved through the aggregation of features from multiple layers, which helps in capturing finer details even when objects are partially obscured or under varying illumination. Prediction heads refine the detections and produce the final output. YOLOv8 achieves real-time performance by optimizing network architecture and leveraging techniques such as feature pyramid networks and anchor boxes.
5. YOLOv8 variants and model selection: The YOLOv8 architecture offers a spectrum of variants tailored to meet specific requirements, providing scalability and memory efficiency for diverse applications. These variants range from large models suitable for intricate feature extraction to nano or small models optimized for constrained computational resources, enabling seamless integration into autonomous vehicle systems where memory and processing power are limited.
  In the context of YOLOv8 models, three key metrics parameters (params), FLOPs (floating point operations), and CPU ONNX speed are commonly used to describe a model’s computational complexity and performance, as shown in Table 1.
  (a) Parameters (params): The number of trainable weights in a model. More parameters increase learning capacity, thus requiring more memory and computational power.
  (b) FLOPs (floating point operations): The total number of mathematical operations required for inference. Higher FLOPs indicate more computational complexity, leading to better accuracy but slower speed.
  (c) CPU ONNX speed: The inference time (in ms) of a model on a CPU using the ONNX format.
  (d) A100 TensorRT speed: It refers to the rapid inference achieved by optimizing models with TensorRT on NVIDIA’s A100 GPU.
  In practical applications, the choice of YOLOv8 variant is crucial for achieving optimal performance while adhering to resource constraints. While large and medium variants may exhibit high training accuracy owing to their capacity to capture complex features, the performance in terms of testing accuracy, particularly precision, can vary significantly across different variants. Surprisingly, experiments reveal that nano and small models often outperform their larger counterparts, especially in real-life scenarios encountered on Indian roads.
  Real-life scenario testing serves as a robust validation of the performance of YOLOv8 variants. These evaluations encompass diverse conditions, including varying weather, traffic patterns, and lighting conditions, mirroring the complexities of real-world driving environments. Notably, nano and small models consistently demonstrate superior performance in terms of accuracy and precision during these tests, showcasing their effectiveness in practical autonomous driving applications.
  In essence, the selection of YOLOv8 variants is a critical consideration in optimizing the balance between model complexity, memory efficiency, and performance in real-world scenarios. By strategically choosing the appropriate variant based on specific requirements and constraints, autonomous vehicle systems can achieve efficient and reliable object detection and classification while minimizing computational overhead.
6. Bounding box visualization: Detected objects are visualized by overlaying bounding boxes on the original frames. Additionally, the confidence scores associated with each detection are displayed alongside the bounding boxes, providing insights into the reliability of the detections.
7. Output generation: The processed frames, with bounding boxes and confidence scores having a threshold value of 0.25 overlaid, are aggregated into a final output format. This could involve generating annotated images or reconstructing the frames into a video with visual annotations.

### 3.4. Training

The training process commenced with initializing the selected YOLOv8 models using pretrained weights from the COCO dataset [37]. Transfer learning was employed to adapt the models to the specific characteristics of Indian road scenes, leveraging the knowledge learned from the COCO dataset.

The training process involved several steps:

- Fine-tuning: The initialized models were fine-tuned on the improved Dats_2022 dataset using gradient descent-based optimization algorithms. Hyperparameters such as learning rate, batch size, and regularization techniques were carefully tuned through iterative experimentation to optimize model performance.
- GPU acceleration: Training was performed on GPU-accelerated hardware to expedite convergence and reduce training times, enabling faster iterations and model refinement.

Several variants of YOLOv8 were trained with different numbers of epochs; the details are as follows:

1. YOLOv8-Nano for 10 epochs.
2. YOLOv8-Nano for 20 epochs.
3. YOLOv8-Nano for 100 epochs.
4. YOLOv8-Small for 30 epochs.
5. YOLOv8-Small for 100 epochs.
6. YOLOv8-Medium for 30 epochs.
7. YOLOv8-Large for 10 epochs.

### 3.5. Evaluation Standards

#### 3.5.1. Experimental Setup

The experimental setup for evaluating the proposed YOLOv8-based object detection and classification system in autonomous vehicles involved the utilization of Google Colab for cloud-based computing resources. This section outlines the details of the software environment and computing resources employed during the experimentation process.

- Software environment: The software environment comprised the Ultralytics repository, a deep learning framework specifically tailored for object detection tasks, and associated libraries to support the development and deployment of the YOLOv8-based system. The software components utilized in the experimental setup included the following:
  1. Ultralytics repository: Utilized for training and inference with YOLOv8 architecture.
  2. Python programming language: Python 3.10.
  3. OpenCV library: OpenCV 4.5.3.
  These software tools and libraries provided a robust development environment for training the YOLOv8 model, conducting experiments, and evaluating the performance of the object detection system within the Google Colab environment.
- Hardware environment: The hardware environment comprises the webcam and CPU used during live testing of the trained model on Indian roads. Given below are the specifications of each item used:
  1. Camera: ASUS Webcam C3 (Asus India)—1080p and 60 fps.
  2. CPU: 11th Gen Intel Core i7-1165G7 Processor (Intel India)—4 cores and 8 logical processors.

#### 3.5.2. Performance Metrics

The performance of each trained YOLOv8 variant was evaluated using comprehensive testing protocols to assess detection accuracy and classification performance. The following metrics were computed:

1. Precision, recall, and F1-score: Metrics used to measure the model’s ability to detect and classify objects accurately.
2. Qualitative analysis: In addition to quantitative metrics, qualitative analysis was conducted to evaluate the models’ performance in real-world scenarios. This involved visually inspecting the model’s predictions and assessing its ability to detect and classify obstacles accurately in diverse environments.

### 3.6. Optimization and Deployment

After satisfactory performance evaluation, the selected YOLOv8 variant underwent optimization for deployment in real-time applications. This optimization process involved the following.

#### 3.6.1. Model Compression

Techniques such as quantization and pruning were applied to reduce the model’s memory footprint and inference latency without sacrificing performance.

#### 3.6.2. Integration

The optimized model was integrated into a real-time obstacle detection and classification system tailored for deployment in autonomous vehicles operating on Indian roads. A pretrained YOLOv8 model, optimized for vehicle detection, forms the core of the real-time system.

Integrated with Flask, a lightweight web framework, it creates the backend for a live web application. Flask efficiently manages video streams captured using OpenCV’s camera access functionalities. Each frame is routed to the YOLOv8 model within the application, enabling real-time detection and bounding box generation for identified vehicles. These processed frames are then streamed back to the user’s web browser for visualization. Leveraging a standard camera prioritizes cost-effectiveness, making the system suitable for resource-limited deployments. Further optimization strategies, like model quantization and WebRTC for video streaming, could be explored for enhanced performance. When deploying this system in real-world scenarios, addressing privacy concerns through techniques like anonymization is crucial.

### 3.7. Iterative Refinement

Throughout the methodology, an iterative approach was adopted to refine the models and system components based on insights gained from experimentation and evaluation. Feedback from testing and validation phases informed adjustments to data preprocessing, model architecture, and training strategies, ensuring that the final system was robust and well-adapted to the challenges of real-time obstacle detection and classification in autonomous vehicle navigation on Indian roads.

1. Experimentation and evaluation: Initial YOLOv8 models and system components are built and tested with various configurations.
2. Testing and validation: The system is rigorously tested against datasets and real-world scenarios to assess accuracy, speed, and robustness.
3. Feedback collection: Insights, challenges, and areas for improvement are identified from testing and validation.
4. Analysis and insights: Feedback is analyzed to pinpoint key areas requiring refinement.
5. Adjustments and refinements: The system is adjusted based on the analysis, including hyperparameter tuning, data preprocessing modifications, model architecture optimization, or training strategy adjustments.
6. Iterative testing and validation: The refined system is retested and validated to measure the impact of adjustments. This loop continues until satisfactory performance is achieved.
7. Performance monitoring: Performance metrics are continuously monitored throughout the process to track progress and identify any issues.
8. Deployment and continuous improvement: Once a satisfactory system is developed, it is deployed for real-world use. However, the refinement process is ongoing to ensure the system remains adaptable and effective over time.

## 4. Experimental Evaluation

### 4.1. Object Classification Performance

The training metrics for different model variants are presented in Table 2. Across various configurations, the model’s precision, recall, and F1-score exhibit notable variations. In the “Large” variant trained for 10 epochs, precision stands at 0.31, with recall and F1-score at 0.2, indicating modest performance. Conversely, the “Medium” variant trained for 30 epochs displays significantly improved metrics, with precision reaching 0.79, recall at 0.93, and F1-score at 0.82, suggesting robust classification capabilities.

The “Small” variant trained for 30 epochs yields a precision of 0.5, recall of 0.15, and F1-score of 0.15, indicating a considerable drop in performance compared to previous configurations. Increasing the training duration to 100 epochs for the “Small” variant notably enhances performance, with precision and recall reaching 0.8 and 0.97, respectively, resulting in an F1-score of 0.89, signifying significant improvement.

Examining the “Nano” variants, which likely represent models designed for resource-constrained environments, it can be seen that the performance metrics vary. The “Nano” variant trained for 10 epochs exhibits a precision of 0.65 and a recall of 0.23, indicating a trade-off between precision and recall compared to previous configurations. Further, the “Nano” variant for 30 epochs enhances performance, achieving a precision of 0.65, recall of 0.4, and F1-score of 0.41.

It can be said that the best model is the “Nano” variant trained for 20 epochs because of its performance as well as the trainable parameters, i.e., 3.2 million. Even comparing it with “NITCAD—Developing an object detection, classification and stereo vision dataset for autonomous navigation in Indian roads” results, i.e., a precision of 0.84 over 6 classes, this model performs really well for having a precision of 0.65 over 35 classes. Overall, these results highlight the impact of model architecture, training duration, batch size, and fine-tuning on the performance of object detection models, providing insights for optimizing model configurations tailored to specific use cases and resource constraints.

Table 3 provides a comprehensive comparison of various YOLO models across different variants and metrics, including precision, recall, and F1-score for both training and testing phases. Notably, YOLOv8 models exhibit remarkable performance improvements compared to their predecessors. Specifically, YOLOv8 Small demonstrates significant enhancement in training metrics, achieving a precision of 0.72 and recall of 0.88, although its test performance shows room for improvement. YOLOv8 Nano also stands out with a balanced precision of 0.65 and recall of 0.50, translating to a higher F1-score of 0.41 on training data and 0.30 on testing data, outperforming many other variants in the test phase. These results underscore YOLOv8’s superior efficacy and robustness, making it a compelling choice over other state-of-the-art models in the YOLO series.

### 4.2. Object Detection Performance

**Figure 6:**
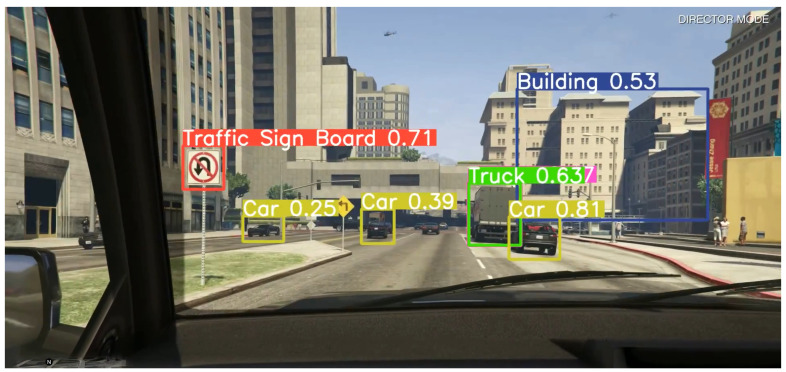
Syntactic testing data.

During the analysis of the model for performance with syntactic data, it was observed that the model detected all the objects, and most of them were classified correctly. Even though there were some misclassifications, the performance can be considered as excellent because the training was performed in an Indian setting, whereas the simulation it was tested on is more of an international setting. This showcases the flexibility of the model and the fact that it is not limited to just Indian roads. A sample image in Figure 6 from the video the model was tested upon is showed below.

During the thorough analysis of the performance of real-time object identification, a number of situations with changing traffic and ambient circumstances were examined. The goal was to evaluate the detection model’s resilience and reliability in various settings by utilizing a camera configuration. The best testing model was used, i.e., YOLOv8-Nano Variant trained for 20 epochs for the detection performance analysis. The confusion matrix of the model is complex but determines the fact that all the classes are detected and classified with utmost precision, and this fact is backed up by the real-time analysis of the model performance as shown later.

**Figure 7:**
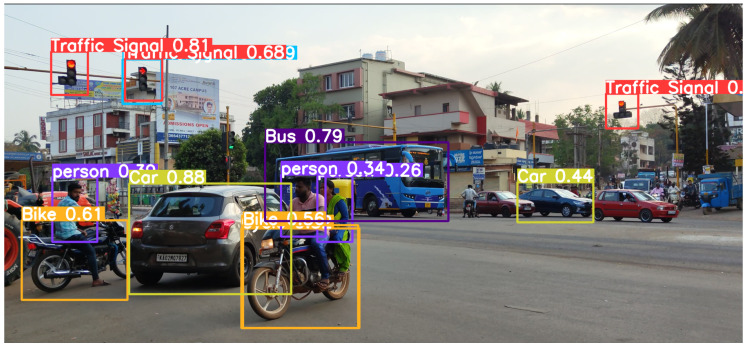
Overlapping condition.

First, it can be seen in Figure 7 how “Overlapping” conditions affect the precision of object detection. The model showed respectable recall and precision in conditions where vehicles overlap with each other. However, as distance is increased, it does not seem to detect objects like some cars. It does not seem to have any problem detecting a particular class, but the distance is surely a factor to consider here.

**Figure 8:**
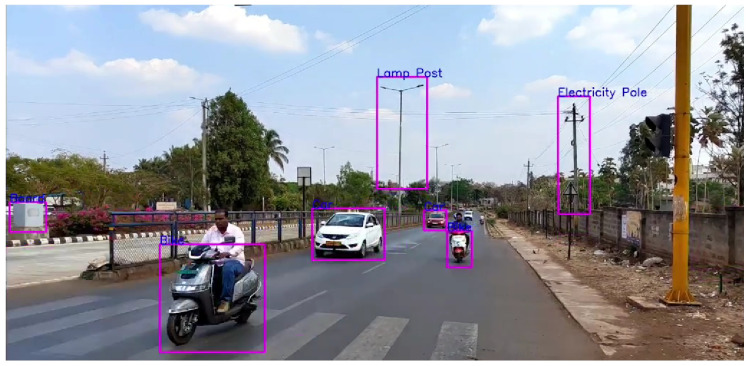
Distance of 10–50 m.

It can be seen in Figure 8, for a range of 10–50 m, that all the detections and classifications such as Car, Bike, and Lamp Post are correct except for one misclassification.

**Figure 9:**
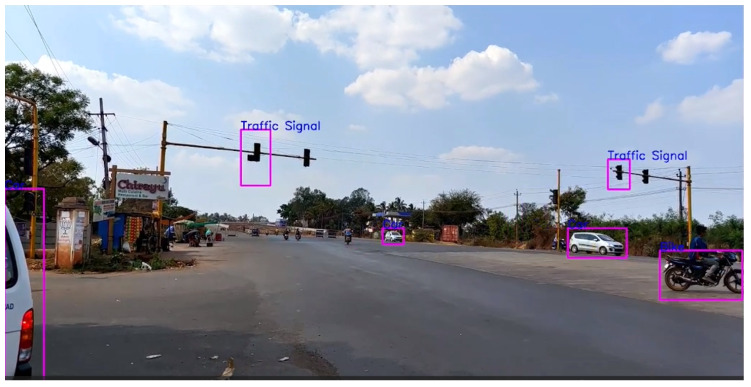
Distance of 10–100 m.

As for the range of 10–100 m in Figure 9, all the detections are correct, but as the distance increases, the model is not able to detect objects.

**Figure 10:**
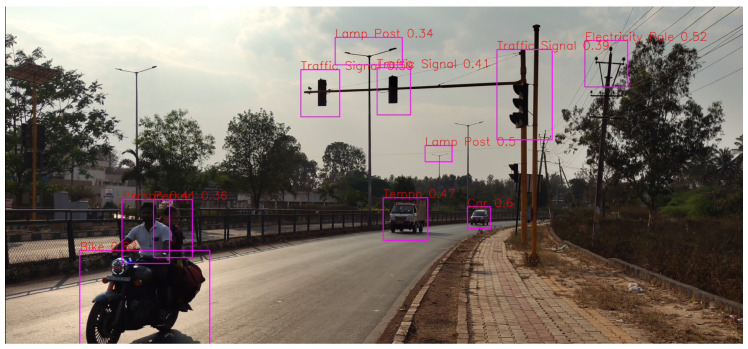
Distance condition.

It can be seen how “Distance” affects the effectiveness of object detection in Figure 10. When objects were in the range 10–50 m, the model demonstrated impressive precision. Almost all the objects seem to be detected and classified correctly. In object detection for the range of 10–100 m, the model’s accuracy did, however, decrease as the distance between the camera and objects increased. Some objects were not detected, but there seems to be no misclassifications.

Furthermore, the distance here is calculated relatively, based on the size of bounding boxes surrounding the object. So, in looking at the size of bounding boxes, it can be inferred that as distance increases, meaning the box size decreases, the accuracy decreases.

The effect of environmental elements like “Weather” conditions was also looked at to see how it affected the accuracy of object detection. The model proved resilient in inclement circumstances, including rain and fog.

**Figure 11:**
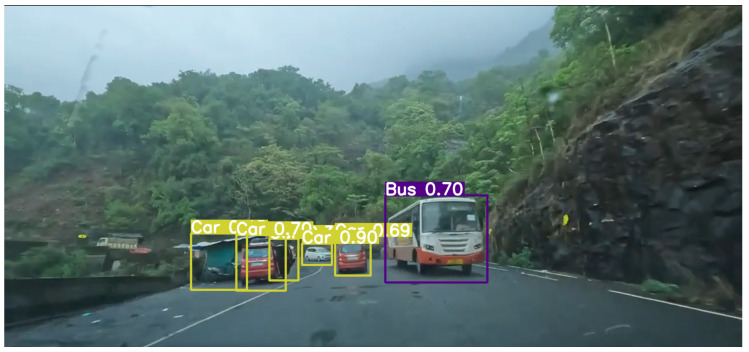
Rainy condition.

It can be seen in Figure 11 that all objects present are getting detected but not all detected objects are classified correctly, for example, along with all the cars, a tent is getting detected and classified as a car. This could be due to the overlapping behavior of objects in this scenario or due to the fact that “Car” class is the predominant one on roads.

**Figure 12:**
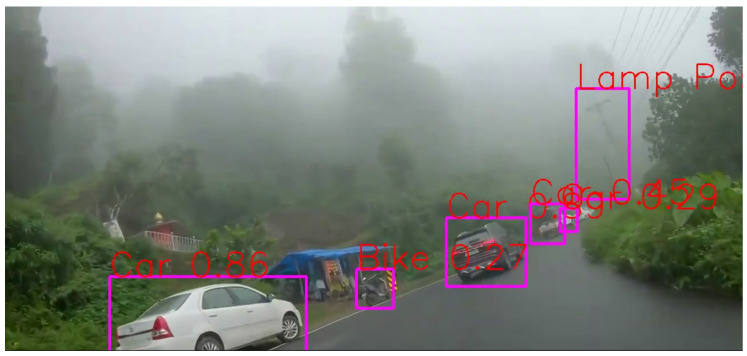
Misty condition.

It can be seen in Figure 12 that even in misty conditions, the model predicts everything correctly. There is a lot of uncertainty in the image, but the result is really good. There seem to be no misclassifications, but one thing to note here would be the low confidence score for the detected objects.

Additionally, assessment of the model’s performance in scenarios with “Light Traffic”, “Medium Traffic”, and “Dense Traffic” situations was carried out. The model’s accuracy held up well in circumstances with heavily packed traffic, in the day, and at night when light is limited. On the other hand, the model performed consistently in scenarios with less traffic during daytime and medium traffic during nighttime, respectively.

**Figure 13:**
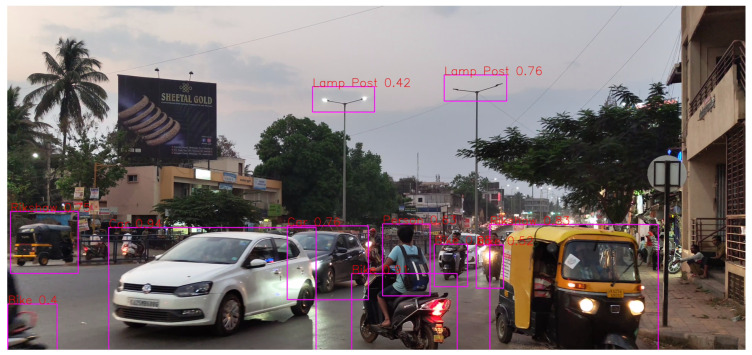
Dense traffic (day).

It can be seen in Figure 13 that the model is able to detect most of the objects and classify them correctly. As the distance and overlapping increases, we can see that some objects get detected and the rest are undetected.

**Figure 14:**
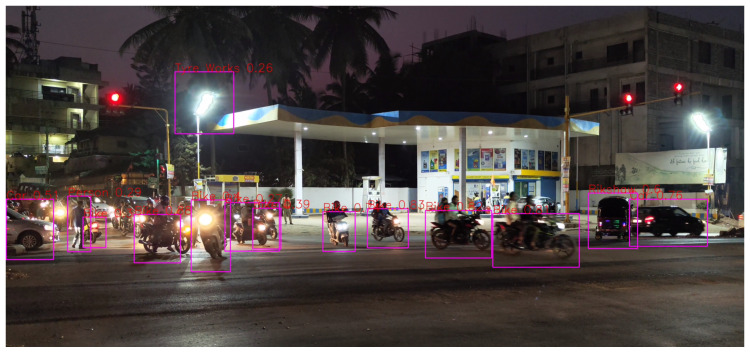
Dense traffic (night).

It can be seen in Figure 14 that the model is able to detect almost all of the objects and classify them correctly, but it missed traffic signals and has a misclassification. The reason for it could be the increased complexity because of distance and low brightness.

**Figure 15:**
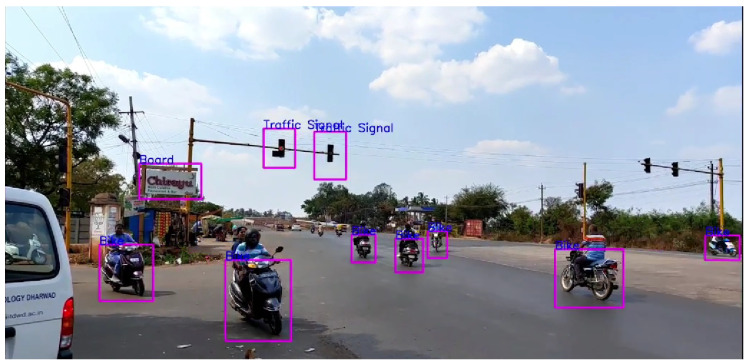
Low traffic (day).

It can be seen in Figure 15 that all the objects, even distant, are detected and classified correctly except for the traffic signals in the distance. For traffic conditions, distance seems to be a relevant factor for object detection and classification, more so than any other condition.

**Figure 16:**
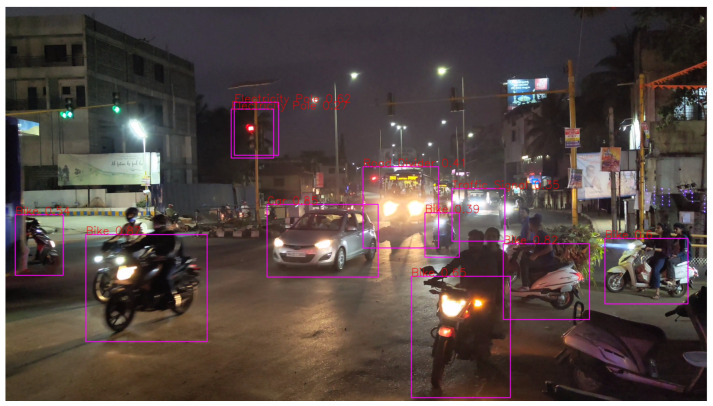
Medium traffic (night).

It can be seen in Figure 16 that objects like Cars and Bike are detected and classified correctly, but there are misclassifications due to it being nighttime and having low visibility. As per the observations before, distance seems to be playing a role here.

All things considered, the results highlight the detection model’s adaptability and efficacy in a variety of real-world circumstances, from changing ambient parameters to dynamic traffic situations. These findings offer insightful information for implementing object detection systems in real-time applications, guaranteeing dependability and excellent performance in a variety of scenarios.

## 5. Discussion

Previous object detection models include algorithms like Faster R-CNN and SSD, but YOLOv8 turns out to be the best with this problem statement. What we want to achieve is that the objects get detected accurately in the Indian roads setting, and YOLOv8 has certain advantages over the previous methods used by many other object detection models.

Table 4 shows the comparison between the top five Indian datasets and the dataset used here. It can be seen that the most number of classes as well as diverse conditions like traffic and weather along with the pedestrian classes, which are the most important classes in an Indian roads setting, are included in the dataset used for this paper, i.e., DATS_2022 Dataset. This dataset has a variety of vehicle and background images as well as images added for making the distribution less skewed and to give more importance to certain classes. Hence, it can be said that this dataset would be the best to capture the essence of Indian roads and give the best accuracy if the model is trained on it.

To strengthen the discussion, it is important to clarify the research gaps addressed and highlight the scientific contributions of this work in relation to prior research. One major gap addressed by this study is the lack of a dedicated dataset tailored to the diverse and unpredictable conditions of Indian roads. While existing models like YOLOv7 and Fast-RCNN have shown high performance on standard datasets, they often fall short in environments characterized by irregular traffic patterns, diverse weather conditions, and the frequent presence of pedestrians, which are common in the Indian context. By leveraging the DATS_2022 dataset, which includes a comprehensive range of object classes and conditions specific to Indian roads, this research directly fills this gap and provides a more realistic and effective solution for obstacle detection in such environments.

Furthermore, while prior research has often relied on complex point cloud data processing from LIDAR or RADAR sensors, this paper proposes a more accessible approach using only camera-based visual data, significantly simplifying the system architecture while maintaining robust detection capabilities. This research contributes scientifically by demonstrating that accurate real-time obstacle detection and classification can be achieved over a wider range of object classes and environmental conditions without the need for expensive sensor setups. The versatility and flexibility of the proposed model, combined with its ability to generalize across varied and challenging conditions, offer a valuable step forward in advancing autonomous vehicle technologies, particularly for developing regions like India where such technologies have been less explored.

Despite the promising results and the suitability of the DATS_2022 dataset for capturing the complexities of Indian roads, the proposed method has certain limitations that must be addressed. One of the main challenges is the relatively lower recall rate of 0.5, which indicates that the model may still miss detecting some objects in certain scenarios, especially in extreme lighting conditions or when objects are partially occluded. Additionally, while the model is designed to handle a diverse range of classes, its accuracy could potentially drop when exposed to rare or highly unusual objects that are underrepresented in the dataset. Another limitation is the reliance solely on visual data from cameras, which may not be sufficient in low-visibility conditions such as heavy rain, fog, or night-time driving, where other sensory inputs like LIDAR or RADAR might offer complementary information. Lastly, while the method simplifies object detection by using bounding boxes, more advanced techniques like 3D bounding boxes or depth estimation could further enhance obstacle detection and improve overall safety in complex traffic environments. These limitations present opportunities for future improvements to ensure more robust and reliable performance in real-world autonomous driving scenarios.

## 6. Conclusions and Future Work

In conclusion, this paper introduces a novel approach to object detection for autonomous vehicles, leveraging the robust YOLOv8 architecture to provide real-time obstacle avoidance. By utilizing camera-based image sensors, the model offers a cost-effective, scalable solution that reduces the dependency on expensive sensors like LIDAR, without compromising detection accuracy. This work represents a significant step forward in simplifying the integration of obstacle detection systems into existing autonomous platforms, lowering barriers to adoption while maintaining high performance across diverse road conditions, particularly on complex Indian roads.

The broader impact of this research lies in its potential to reshape the landscape of autonomous driving technology. By focusing on a camera-only detection system, this approach makes autonomy more accessible for regions with infrastructural and environmental challenges, contributing to the global push for safer, more reliable, and affordable autonomous vehicles. Furthermore, this study opens new avenues for future research, particularly in integrating camera-based detection systems with other sensor modalities to further enhance accuracy in adverse conditions. The advancements presented here not only improve the current state of the field but also pave the way for future innovations in autonomous navigation, with a clear focus on real-world applicability and scalability.

In future work, an interesting avenue for exploration could involve comparing the performance of camera-based systems with LiDAR-based systems for object detection and classification in autonomous vehicles. This comparison could shed light on the strengths and weaknesses of each technology and provide insights into their complementary roles in enhancing the perception capabilities of autonomous vehicles.

## Figures and Tables

**Table 1:** YOLOv8 variants comparison.

Variant	Speed (ms) CPU ONNX	Speed (ms) A100 TensorRT	Parameters (M)	FLOPs (B)
Nano	80.4	0.99	3.2	8.7
Small	128.4	1.20	11.2	28.6
Medium	234.7	1.83	25.9	78.9
Large	375.2	2.39	43.7	165.2

**Table 2:** Evaluation study.

Variant	Epochs	Trainable Parameters	Precision (Train)	Recall (Train)	F1-Score (Train)	Precision (Test)	Recall (Test)	F1-Score (Test)
Large	10	43.7 m	0.64	0.54	0.54	0.31	0.20	0.20
Medium	30	25.9 m	0.79	0.93	0.82	0.30	0.16	0.16
Small	30	11.2 m	0.72	0.88	0.80	0.50	0.15	0.15
Small	100	11.2 m	0.80	0.97	0.89	0.25	0.20	0.15
Nano	10	3.2 m	0.65	0.23	0.23	0.50	0.20	0.15
Nano	20	3.2 m	0.65	0.40	0.41	0.60	0.50	0.30
Nano	50	3.2 m	0.65	0.62	0.60	0.23	0.13	0.13

**Table 3:** Comparison with state-of-the-art models.

Model	Variant	Epochs	Precision (Train)	Recall (Train)	F1-Score (Train)	Precision (Test)	Recall (Test)	F1-Score (Test)
YOLOv5	Small	20	0.56	0.10	0.17	0.50	0.09	0.15
YOLOv5	Nano	20	0.59	0.09	0.15	0.53	0.13	0.21
YOLOv7	-	20	0.62	0.24	0.35	0.52	0.11	0.19
YOLOv7	Tiny	20	0.65	0.26	0.37	0.58	0.23	0.23
YOLOv8	Small	30	0.72	0.88	0.80	0.50	0.15	0.15
YOLOv8	Nano	20	0.65	0.40	0.41	0.60	0.50	0.30

**Table 4:** Top five Indian datasets comparison with this dataset.

Dataset Name	No. of Classes	Diversity	Pedestrians
DATS_2022	35	Yes	Yes
Indian Vehicle Image [38]	11	Yes	No
YOLOv8 Indian Roads [39]	10	No	Yes
Vehicle Detection [40]	8	No	No
IDD	34	Yes	Yes
IRUVD [41]	14	Yes	Yes

## Data Availability

GitHub repository link—https://github.com/AryanTN05/obstacle-detection-and-classification (accessed on 12 August 2024).

## Abbreviations

The following abbreviations are used in this manuscript:

YOLO V8	You Only Look Once Version 8
GSI	GPU-friendly Subgraph Isomorphism
LiDAR	Light Detection Furthermore, Ranging
IDD	Indian Driving Dataset
NITCAD	National Institute of Technology Calicut Autonomous Driving
RCNN	Region-Based Convolutional Neural Network
SSD	Single-Shot Multi-box
DATS	Driver Assistance System
TLA	Three-Letter Acronym
LD	Linear Dichroism
